# Ultrahigh Voltage Electron Microscopy Links Neuroanatomy and Neuroscience/Neuroendocrinology

**DOI:** 10.1155/2012/948704

**Published:** 2011-12-08

**Authors:** Hirotaka Sakamoto, Mitsuhiro Kawata

**Affiliations:** ^1^Laboratory of Neuroendocrinology, Ushimado Marine Institute, Graduate School of Natural Science and Technology, Okayama University, Kashino, Ushimado, Setouchi, Okayama 701-4303, Japan; ^2^Department of Anatomy and Neurobiology, Kyoto Prefectural University of Medicine, Kawaramachi-Hirokoji, Kamigyo-ku, Kyoto 602-8566, Japan

## Abstract

The three-dimensional (3D) analysis of anatomical ultrastructures is extremely important in most fields of biological research. Although it is very difficult to perform 3D image analysis on exact serial sets of ultrathin sections, 3D reconstruction from serial ultrathin sections can generally be used to obtain 3D information. However, this technique can only be applied to small areas of a specimen because of technical and physical difficulties. We used ultrahigh voltage electron microscopy (UHVEM) to overcome these difficulties and to study the chemical neuroanatomy of 3D ultrastructures. This methodology, which links UHVEM and light microscopy, is a useful and powerful tool for studying molecular and/or chemical neuroanatomy at the ultrastructural level.

## 1. Introduction

The three-dimensional (3D) analysis of anatomical ultrastructures is extremely important in most fields of biological research. However, it is considerably difficult to perform a 3D image analysis of exact serial sets of ultrathin sections. Although 3D reconstruction from ultrathin sections (~100 nm thickness) has been generally used to obtain 3D information, this technique is applicable only for small specimen areas because of the technical and physical difficulties under the transmission electron microscopy, restricted to approximately 1 mm^2^ area. On the other hand, due to tremendous development of various techniques in molecular biology (e.g., green fluorescent proteins and their color variants), as well as the development of live imaging techniques, the structure of biological molecules and their functional changes are calculated and visualized in 3D at subnanometer resolution [1, 2]. With the aid of confocal laser scanning microscopy, it is now possible to image and quantify the 3D organization of these cell processes; however, the detailed morphology of the complicated terminal processes of these cells remains obscure because of the insufficient spatial resolution of light microscopy and visualization methods that depend on fluorescence [3–6]. In addition, unstained domains are very difficult to recognize [3]. In contrast, conventional transmission electron microscopy provides extremely detailed and fine structural information, but the images obtained are mostly 2D due to the physical properties of this imaging technique (use of ultrathin sections). Consequently, it is too difficult to relate electron micrographs to the 3D structures of cells.

The high penetration power of electrons at an ultrahigh accelerating voltage enables the examination of thick sections of biological specimens. This property of ultrahigh voltage electron microscopy (UHVEM) is particularly useful for the morphological study of the central nervous system [7]. Differences in behavioral neuroendocrinology (i.e., control of reproduction, sexual behavior, and food intake) are commonly correlated with differences in neuroanatomy. In the central nervous system, a population of synaptic inputs from a brain nucleus can influence axosomatic synapses whereas the structural analysis of multiple major synaptic inputs into the dendrites located outside the nucleus is difficult to perform methodologically. However, using UHVEM we overcame these difficulties and described a powerful methodology to study the chemical neuroanatomy of 3D ultrastructures [8, 9]. Here, 3D analysis of neuroanatomy at the ultrastructural level using UHVEM is summarized.

## 2. Golgi Impregnation

The gross morphology of neurons and glial cells have been described in detail using light microscopy in combination with various metal impregnation techniques such as Golgi silver staining [10]. The Golgi method is very useful and has been utilized by many neuroanatomists over the past century. Subsequently, the rapid Golgi impregnation procedure, a newly developed method, is also applied in qualitative and quantitative characterization of neuronal morphology analyses both at light and electron microscopic levels [11–14]. We used rapid Golgi impregnation, in combination with UHVEM to visualize neuronal structures at the electron microscopic levels.

Rats were overdosed with sodium pentobarbital (100 mg/kg body weight), and perfusion fixed using 2.5% paraformaldehyde and 2.5% glutaraldehyde in a 0.1 M phosphate buffer (PB; pH 7.4). Brain sections (100–300 *μ*m thickness) were prepared using a microslicer (Dosaka EM, Kyoto, Japan). After the sections were processed using the rapid Golgi technique as described elsewhere [11, 12], the tissues were dehydrated and flat embedded in epoxy resin and preliminarily examined using a light microscope [8]. Appropriately Golgi-impregnated neurons in the hippocampus were selected for the following procedures. Thick sections of selected neurons were remounted for further sectioning. Two to five-micrometer thick sections were cut using an ultramicrotome (Ultracut, Reihert-Jung, Wetzlar, Germany) and collected on copper grids. Each specimen was first photographed using a light microscope for reference purposes, then examined using UHVEM (Hitachi H-1250 M; National Institute for Physiological Sciences, Okazaki, Japan) at an accelerating voltage of 1,000 kV. Stereopaired photomicrographs were prepared using UHVEM by tilting the specimen stage ±8°.

**Figure 1:**
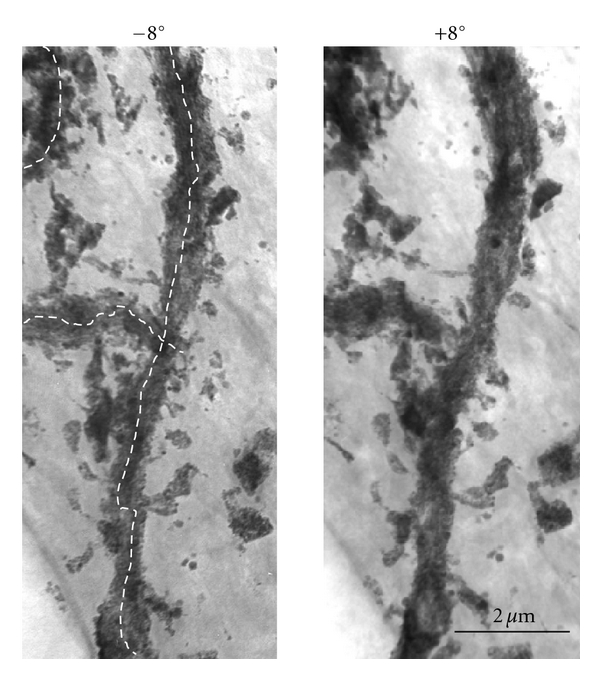
Stereopaired UHVEM images obtained by tilting the specimen stage ±8° to reveal the 3D structure of the impregnated Golgi dendrites of CA1 pyramidal neurons of the hippocampus.

In the hippocampus, excitatory synapses develop very rapidly and most of the components necessary to perform their complex functions can be found during the early postnatal period [15]. Over 90% of the excitatory synapses are formed on small postsynaptic protrusions that are known as “dendritic spines” [16]. Dendritic spines are the major excitatory input sites of the hippocampal pyramidal neurons and are related to learning, memory, functional recovery, and plasticity of the central nervous system [17]. Accordingly, the precise morphometry of these structures is indispensable for a better understanding of neuronal function and useful for modeling neuronal circuitry. A 3D morphometric study was performed using stereopaired UHVEM images. First, nontilted and ±8°-tilted UHVEM images of 4-*μ*m thick specimens were examined. UHVEM stereoscopic analysis of the thick Golgi-impregnated materials revealed that the thorny processes appeared as a bunch of grapes, consisting of long protrusions that were studded with many bulbous appendages (Figure 1). Because dendritic spines are the major excitatory input sites of the neurons in the central nervous system [16], 3D morphometric analysis, in combination with Golgi impregnation and UHVEM stereopaired images, is very useful for elucidating the neural functions of living matter (Figure 1).

## 3. Retrograde Tracing and Immunocytochemistry

Onuf's nucleus, located in the ventral horn of the sacral spinal cord of many mammals, including humans, is a sexually dimorphic nucleus that innervates the perineal muscles that are involved in sexual behavior. In humans, it is a distinct group of neurons located in the ventral part of the anterior horn of the sacral region of the spinal cord involved in the maintenance of micturition and defecatory continence, as well as muscular contraction during orgasm [18]. The number of neurons in Onuf's nucleus is greater in males than in females [18–21]. On the other hand, the spinal nucleus of the bulbocavernosus (SNB) of rats, located in the lower lumbar and upper sacral spinal segments, is homologous to Onuf's nucleus in that it innervates the striated perineal muscles that are attached to the base of the penis [21–23]. The distribution of serotonergic fibers and terminals in this nucleus in rats is also different between the sexes (male dominant) [24–26]. SNB also plays a significant role in male sexual functions in the rat [22, 23, 27, 28]. Male rats have a larger and a greater number of SNB motoneurons than females; this dimorphism results from differences in perinatal androgen signaling through a mechanism mediated by the androgen receptor [22]. On the other hand, we recently reported that a collection of neurons within the upper lumbar spinal cord (L3-L4 level) project axons with gastrin-releasing peptide (GRP) to the lower lumbar spinal cord, controlling male reproductive functions in rats [9, 29, 30]. It has also been reported that the sexually dimorphic distribution of GRP-immunoreactive fibers in the lower lumbar spinal cord is profoundly regulated by circulating androgen levels [31], mirroring changes in SNB motoneuron arborizations and other synaptic populations [23]. However, due to methodological difficulties, no direct evidence has been reported regarding GRP synaptic inputs to the SNB motoneurons. The aim of the current study was to determine the axodendritic synaptic inputs of GRP neurons that project into perineal SNB motoneurons and bulbocavernosus muscles. Immunoelectron microscopy, in combination with a retrograde tracing technique using UHVEM, was employed to visualize the 3D ultrastructures of the central nervous system [8].

 Rats were deeply anesthetized using intraperitoneal injections of 50 mg/kg body weight sodium pentobarbital and bilaterally injected with 1 *μ*L of 0.2% cholera toxin *β* subunit-horseradish peroxidase conjugate (CTb-HRP; List Laboratories, Cupertino, CA, USA) into the bulbocavernosus muscles. Rats were overdosed with sodium pentobarbital (100 mg/kg body weight) 48–96 h after the CTb-HRP injection, and then perfusion fixed with 4% paraformaldehyde, 0.2% glutaraldehyde, and 1.25% picric acid in a 0.1 M PB solution (pH 7.4). Spinal cords were immediately removed and immersed in the same fresh fixative for 3 h. Spinal sections (L5-L6 level; 30 *μ*m thickness) were prepared using a microslicer (Dosaka EM). Next to visualize SNB somata and dendrites, retrogradely labeled with CTb-HRP, the tetramethylbenzidine (TMB)/diaminobenzidine- (DAB-) nickel method was performed as previously described [32]. Sodium tungstate was used as the stabilizer. Sections were then placed in a 0.1 M PB with 25% sucrose and 10% glycerol for 1 h for cryoprotection, then freeze-thawed using liquid nitrogen to enhance the penetration of the antibodies. After blocking with phosphate buffered saline (PBS; pH 7.4) containing 0.05% Triton X-100, 1% normal goat serum, and 1% BSA for 2 h, the sections were incubated in rabbit anti-GRP serum (Phoenix Pharmaceuticals, Burlingame, CA, USA) at a 1 : 5,000 dilution in the blocking solution, for 5 days at 4°C. After washing with PBS, the sections were treated with biotinylated goat anti-rabbit IgG (Nichirei, Tokyo, Japan), at a 1 : 1,000 dilution in the blocking solution for 3 h at room temperature. After washing with PBS, GRP immunoreactivity was developed using the streptavidin-biotin-HRP complex/DAB-nickel method, as previously described [29, 33]. After washing with 0.1 M PB, the sections were placed in a 0.1 M PB solution with 1% OsO_4_ for 90 min, dehydrated, and flat embedded in epoxy resin. The embedded sections were viewed using an Olympus Optical BH-2 microscope (Tokyo, Japan), and regions that may contain GRP synapses were selected. These sections were further cut into serial 2 *μ*m-thick semithin sections and collected on copper grids coated with collodion film. Each specimen was first photographed using a light microscope for reference purpose, then examined using UHVEM at an accelerating voltage of 1,000 kV and conventional transmission electron microscopy (EM; JEM-1220, JEOL, Tokyo, Japan) at an accelerating voltage of 80 kV. Stereopaired photomicrographs using UHVEM were prepared by tilting the specimen stage ±8°. Neuronal profiles were identified using the criteria described by Peters et al. [34].

 Retrograde tracing is a reliable neuroanatomical method used to locate the somata and dendrites of the motoneurons that innervate the somatic muscles [29, 32, 35]. Neurons, retrogradely labeled with CTb-HRP, were visualized by the TMB method, and were observed only in the SNB at the fifth and sixth lumbar segments of the spinal cord. Using light microscopy, we found densely stained somata and dendrites that were diffusely distributed, or in a dot-like fashion, and often appeared to make close appositions with immunolabeled GRP-immunoreactive axons. These appositions were particularly abundant in the dorsal gray commissure (DGC). GRP-immunoreactive somata were not observed in the SNB.

**Figure 2:**
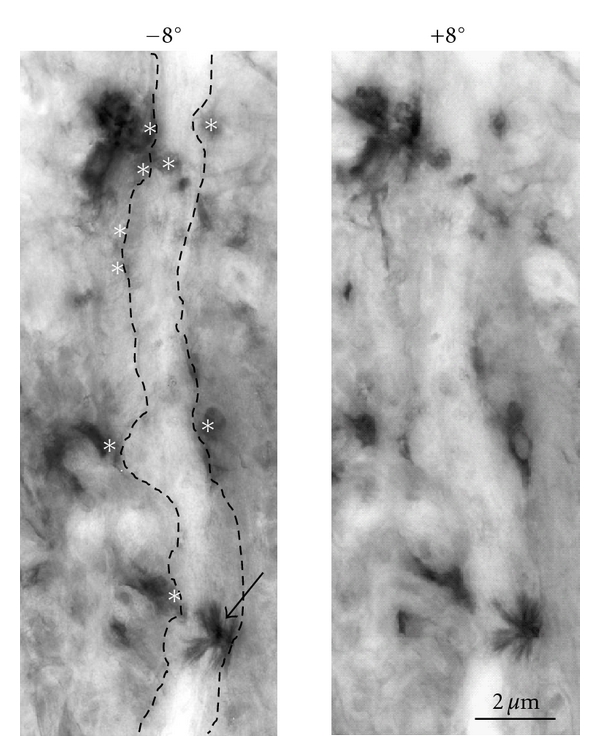
Stereopaired UHVEM images obtained by tilting the specimen stage ±8° reveal the 3D structure of the axonal projections (white asterisks) of a single SNB dendrite. The arrow indicates an electron-dense TMB reaction product of an SNB dendrite with the characteristic radial structure. This figure was reproduced from the research by Sakamoto et al. [8] with permission.

 In terms of their ultrastructures, the TMB reaction products in the CTb-HRP-labeled SNB neurons were also diffusely distributed within the cytoplasm, as revealed by UHVEM and conventional EM [8, 32]. The TMB reaction products were more electron-dense than the DAB reaction products of the GRP-immunoreactive axons [8]. The TMB reaction products were electron-dense small bodies, with a 3D radial spindle-like shape in clusters of various sizes within the cytoplasm and dendrites (Figure 2). The DAB reaction products were visualized as fine, fuzzy materials that were homogeneously distributed throughout the axons and terminals of the GRP neurons (Figure 2). Strikingly, the 3D TMB crystalline structures were clearly observed by UHVEM and were easily distinguishable from the DAB reaction products (Figure 2). Interestingly, 3D analysis with a polarizing lens revealed that some populations of the GRP-immunoreactive terminals possibly formed synaptic contacts with the SNB motoneurons [8]. GRP-immunoreactive axons were also found to wind around a single distal dendrite within the DGC. Taken together, these results suggest that GRP-containing afferents to SNB motoneurons regulate male sexual reflexes *via* these synapses, since the contraction of bulbocavernosus muscles is involved in penile erection [36].

Conventional EM method revealed synaptic inputs into the SNB motoneurons that innervate the bulbocavernosus muscles [8]. In the analysis of ultrathin serial sections we identified a single synaptic GRP input to a TMB-labeled dendrite; however, multiple inputs were not identified. Conventional EM analysis of the ultrathin serial sections (60-nm thick) only revealed a single synaptic input even though both GRP-immunoreactive fibers and SNB motoneuron dendrites are broadly distributed throughout the DGC. By studying the stereopaired UHVEM images, fine, 3D, axonal projections of GRP neurons to CTb-labeled SNB dendrites were observed in a wide but discrete area. Since the semithin sections (2 *μ*m thick) were first visualized using light microscopy and then analyzed using UHVEM (Figure 2), this report is able to definitively conclude that the ability of UHVEM to detect synaptic formations is superior to conventional EM.

## 4. Conclusions

Here, we summarize the 3D analysis of neuroanatomy at the ultrastructure level using UHVEM. Both UHVEM stereoscopy and morphometry are useful for elucidating the functions of living matter. These techniques can easily be combined with Golgi impregnation, conventional neurotracing, and/or immunoelectron microscopic methods to reveal the fine details of 3D neuroanatomy. In conclusion, we believe that this mixed methodology, which links UHVEM and light microscopy, is a useful and powerful tool for studying molecular and chemical neuroanatomy at the ultrastructural level.
